# CYP4F2 genetic polymorphisms are associated with coronary heart disease in a Chinese population

**DOI:** 10.1186/1476-511X-13-83

**Published:** 2014-05-20

**Authors:** Changqing Yu, Qingkai Yan, Chunjiang Fu, Weibin Shi, Hongyong Wang, Chunyu Zeng, Xukai Wang

**Affiliations:** 1 Department of Cardiology, Daping Hospital, The Third Military Medical University, 10#Changjiangzhilu, Yuzhong District, Chongqing 400042, People’s Republic of China; 2 Chongqing Institute of Cardiology, 10#Changjiangzhilu, Yuzhong District, Chongqing 400042, People’s Republic of China

**Keywords:** Coronary heart disease, CYP4F2 gene, Single nucleotide polymorphism

## Abstract

**Background:**

To explore the relationship between CYP4F2 gene polymorphism and coronary heart disease (CHD) in a Chinese Han population.

**Methods:**

We selected 440 CHD patients and 440 control subjects to perform a case - control study. Four SNPs (rs2108622, rs3093100, rs3093105 and rs3093135) in CYP4F2 gene were genotyped using polymerase chain reaction - restriction fragment length polymorphism (PCR - RFLP) methods. The genotype and haplotype distributions were compared between the case and the control group.

**Results:**

We found both rs2108622 and rs3093105 in CYP4F2 gene were associated with the risk for CHD (*P* <0.01). Haplotype analysis indicated that GGGT haplotype consisted by rs2108622-rs3093100-rs3093105-rs3093135 was associated with CHD risk (OR = 4.367, 95% CI: 2.241 ~ 8.510; P < 0.001), but GGTA haplotype was associated with decreased risk for CHD (OR = 0.450, 95% CI: 0.111 ~ 0.777; *P* <0.001).

**Conclusion:**

CYP4F2 gene polymorphisms were associated with the risk of CHD in Chinese population.

## Introduction

Coronary heart disease (CHD) is one of the most common diseases to influence the human health. Previous study indicated that the morbidity and mortality of CHD are very high [1]. CHD was a complex polygenic disease resulting from the interaction between genetic variations and environmental factors [2]. Although the exact genetic mechanism is unclear, some new susceptibility gene of the CHD have been reported recently, such as apolipoprotein (Apo) E [3], CYP8A1 [4], C5L2 [5], and interleukin (IL)-1 [6]. Grammer et al. [3] analyzed the association between the apo E genotype, CRP and angiographic coronary artery disease (CAD). The concentration of CRP was similar in patients with stable CAD and in controls, but increased in patients presenting with acute coronary syndromes. In models adjusting for the main confounding variables, the ε2 allele was associated with a lower prevalence of angiographic CAD. Therefore, the authors concluded that the apo E genotype is associated with circulating CRP and risk for CAD. Xie et al. [4] performed a case–control study in a Chinese population and investigated the roles of polymorphisms in the CYP8A1 gene in CHD susceptibility. The authors found that CYP8A1 genetic polymorphisms are associated with CHD in Chinese population. Zheng et al. [5] identified a novel SNP-901G > A in C5L2 gene and found this SNP may be a genetic maker of CHD in the Han and Uygur population. Zhou et al. performed a meta-analysis of 13 independent case–control studies and suggested that there were no associations between IL-1 gene cluster polymorphisms and CHD. However, these genes have not been verified in different ethnicities.

20 - hydroxy eicosane arachidonic acid (20-HETE) is a metabolite of arachidonic acid (AA) produced in the kidney by cytochrome P450 enzymes. 20-HETE has potent actions on renal tubular and vascular function including: vasoconstriction secondary to inhibition of large conductance Ca^2+^-activated K^+^ channels in vascular smooth muscle cells and inhibition of Cl^−^ transport in the thick ascending loop of Henle [7,8]. 20-HETE has also been implicated as a second messenger mediating the inhibitory effects of dopamine, PTH, and angiotensin II on Na^+^-K^+^-ATPase activity and sodium transport in the proximal tubule [9,10]. Some studies have suggested that increased levels of 20-HETE in the renal vasculature may underlie the development of hypertension [11], and, animal experiments showed that 20-HETE can cause vasospasm after hemorrhagic stroke, and it participates in the incidence and the development process of CHD and cerebral infarction [12]. In the rat models with myocardial infarction, when the 20-HETE synthesis was suppressed, infarct size can be reduced [12]. In hypertensive mice models, the increased 20-HETE production can lead to oxidative stress and endothelial cell damage, which results in the increased incidence of CHD [13].

CYP4F2 is the major synthase which can catalyze the arachidonic acid to generate 20-HETE. Recently, Stec et al. demonstrated that a SNP (rs2108622, V433M) in CYP4F2 gene may cause 20-HETE reduction from arachidonic acid [14]. Fava et al. [15] studied the elderly patients with cerebral infarction in Sweden, and they found that V433M mutation in CYP4F2 gene was associated with cerebral infarction in male patients. A study [16] based on Japanese population found that G allele frequency of rs2108622 in male patients was higher than that in the controls. TCG haplotypes composed by rs3093135-rs1558139-rs2108622 is a risk factor for cerebral infarction in men [17]. In addition, Fava et al. [18] also found CYP4F2 M433 carriers had significantly higher levels of waist, triglycerides, BP and a composite sum of MetS phenotypes (MetS score) beside lower HDL-cholesterol respect to V-homozygotes. However, the relation between CYP4F2 genetic polymorphisms with CHD in Chinese population remains unclear. In the present study, we utilized a case–control study to reveal the relationship between CYP4F2 gene polymorphism and CHD.

## Materials and methods

### Ethics

The present study has been performed with the approval of the ethics committee of The Third Military Medical University and is in compliance with the Helsinki Declaration. The informed consents of the study were collected from all the candidate subjects.

### Subjects

A total of 440 hospitalized CHD patients in the Department of Cardiology, Daping Hospital, The Third Military Medical University were enrolled from September 2011 to April 2013. All these CHD patients were unrelated Han Chinese people. The patients were diagnosed CHD according to the criteria which was described previously [19]. Briefly, CHD was defined as the presence of stenosis of more than 50% luminal diameter in at least one significant coronary artery on coronary angiography. Patients with ascertained congenital hypercoagulation, with proven disease limiting life expectance or with cocaine abuse were excluded.

During the same period, 440 persons in medical center of the same hospital were selected as the control group. All these control subjects were Han people whose age and sex were matched with the patient group. The subjects with cerebrovascular disease, neurological diseases, kidney disease, blood disorders, cancer, peripheral vascular disease, and autoimmune diseases were excluded from the control group. These control subjects have no the history of CHD and any symptom of CHD. The clinical characteristics including age, gender, height, weight, blood pressure, lipids profiles, fasting glucose, past medical history, drug history, smoking history, and alcohol history were collected.

### DNA extraction

2 mL of fasting venous blood was taken from antecubital vein and placed in EDTA-containing tubes. The genomic DNA extraction kit (Promega Corporation, United States) was used for DNA extraction from blood samples of the subjects according to the kit’s protocol.

### SNPs selection

There are 740 SNPs for the human CYP4F2 gene listed in the National Center for Biotechnology Information SNP database (http://www.ncbi.nlm.nih.gov/SNP). We also screened the data for the Tag SNPs on the International HapMap Project website (http://www.hapmap.org/). We searched tag SNP using Haploview 4.2 software in human HapMap Project database with the following criteria: r^2^ ≥ 0.8 and minimum gene frequency (MAF) ≥ 0.1. We found four tag SNPs (rs3093135, rs2108622, rs3093100, and rs1558139) in CYP4F2 gene.

### Genotyping

We utilized polymerase chain reaction - restriction fragment length polymorphism (PCR - RFLP) to genotype there 4 SNPs according to the protocol reported previously [20,21]. The primers were designed using the primer design software-primer 5.0. The primers were shown in Table 1. To ensure the results to be verified, we used sequenced genomic DNAs as positive controls in our assays. Of the genotyped samples, 10% were duplicated and there was at least one positive and one negative control per 96-well DNA plate in our assays. The accuracy of the genotyping was determined by genotype concordance between duplicate samples. We obtained 100% concordance between the genotyped duplicate samples for each of the SNPs.

**Table 1:** Primers and PCR conditions of four SNPs

**SNPs**	**Primers**	**Endonuclease**	**Tm (°C)**
rs2108622	F: 5’-ATCAACCCGTTCCCACCT-3’	Pvu II	55
	R: 5’-ACATTGTGCTCCCAGACG-3’		
rs3093100	F: 5’-AGTGCTTACTAGGGAACTGGAG-3’	Apa I	54
	R: 5’-AAGGATTCAATGCAGGCCTGGA-3’		
rs3093105	F: 5’-AGCCCTCCCTGCTCTACCT-3’	Eae I	56
	R:5’-CCCACTCCCTAAGCCTCGT-3’;		
rs3093135	F : 5’-GGCAGGCAGTCATCCACA-3’	Hinf I	52
	R: 5’-CCAAACAGGCCCTCACAT-3’		

### Statistical analysis

For each polymorphism, departure of the genotype distribution from that expected from Hardy-Weinberg equilibrium was assessed using the standard *χ*^2^ test or Fisher’s exact test. Genotype frequencies in cases and controls were compared by *χ*2 tests. The genotype-specific risks were estimated as odds ratios (ORs). In all cases wild type genotype served as a reference group. Based on the genotype data of the genetic variations, we performed linkage disequilibrium (LD) analysis and haplotype-based case–control analysis, using the SHEsis software (http://analysis2.bio-x.cn/myAnalysis.php). In the haplotype-based case–control analysis, haplotypes with a frequency of <0.03 were excluded. Statistical significance was established at P <0.05. In the present study, we performed the case–control study included 440 CHD patients and 440 control subjects. The estimated power is 83.3%.

## Results

### The characteristics of participants

There were no significant difference in the distribution of age, BMI, HDL-C and LDL-C between the CHD group and the control group. However, there were significant differences in hypertension, diabetes, smoking history, DBP, SBP and GLU, TG, TC concentration between CHD group and control group (Table 2).

**Table 2:** Characteristics of the participants population

**Groups**	**N**	**Age (years)**	**BMI Kg/m**^**2**^	**SBP (mmol/L)**	**DBP (mmol/L)**	**Hypertension (n, %)**	**Diabetes (n, %)**	**Smoking (n, %)**	**GLU (mmol/L)**	**TG (mmol/L)**	**TC (mmol/L)**	**HDL-C (mmol/L)**	**LDL-C (mmol/L)**
CHD group	440	58.6 ± 13.5	25.2 ± 3.4	143.6 ± 21.1	88.1 ± 16.4	148 (33.6)	139 (31.6)	105 (23.9)	7.4 ± 3.6	3.1 ± 1.8	5.5 ± 2.2	1.3 ± 0.9	2.6 ± 1.4
Control group	440	58.5 ± 13.2	24.9 ± 3.5	124.2 ± 13.5	78.2 ± 13.2	64 (14.5)	66 (15.0)	75 (17.0)	4.6 ± 1.5	1.5 ± 1.0	4.0 ± 0.9	1.4 ± 0.7	2.4 ± 0.9
*P*		0.322	0.053	<0.001	<0.001	<0.001	0.001	<0.001	<0.001	<0.001	<0.001	0.114	0.123

Note: BMI: Body Mass Index; SBP: Systolic Blood Pressure; DBP: Diastolic Blood pressure; GLU: Glucose; TG: Triglycerides; TC: Total cholesterol; HDL-C: High-density Lipoprotein-C; LDL-C: Low-density Lipoprotein-C.

### Hardy-Weinberg equilibrium

The genotype distribution in all SNPs were in line with Hardy-Weinberg genetic equilibrium in both CHD group and the control group (all P > 0.05, data not shown).

### Genotype and allele frequencies

We found GG genotype frequency of rs2108622 were significantly higher in the CHD patients than that in control group (*P* <0.05). And the G allele frequency was also significantly higher than that in control group (P = 0.011, Table 3). TT genotype of rs3093105 was common in CHD patients than that in control subjects (P = 0.003). And the T allele frequency was also significantly higher than that in control group (P < 0.001, Table 3).

**Table 3:** Distributions of CYP4F2 genotypes (N = 440)

**SNPs**	**Allels (1/2)**	**Groups**	**Genotypes (n, %)**			***P *** **value**	**Allele (n, %)**		**OR (95% CI)**	***P *** **value**
			**1/1**	**1/2**	**2/2**		**1**	**2**		
rs2108622	A/G	Case	34(0.077)	168(0.382)	238(0.541)	0.048	236(0.268)	644(0.732)	0.773(0.629 ~ 0.949)	0.011
		Control	46(0.105)	191(0.434)	203(0.461)		283(0.322)	597(0.678)		
rs3093100	C/G	Case	7(0.016)	94(0.214)	339(0.770)	0.482	108(0.123)	772(0.877)	0.877(0.664 ~ 1.158)	0.357
		Control	12(0.027)	97(0.220)	331(0.752)		121(0.138)	759(0.863)		
rs3093105	T/G	Case	22(0.050)	117(0.266)	301(0.684)	0.003	161(0.183)	719(0.817)	1.617(1.241 ~ 2.107)	<0.001
		Control	10(0.023)	87(0.198)	343(0.780)		107(0.122)	773(0.878)		
rs3093135	A/T	Case:	12(0.027)	93(0.211)	335(0.761)	0.654	117(0.133)	763(0.867)	0.876(0.670 ~ 1.147)	0.337
		Control:	15(0.034)	101(0.230)	324(0.736)		131(0.149)	749(0.851)		

### Haplotype analyses

**Figure 1:**
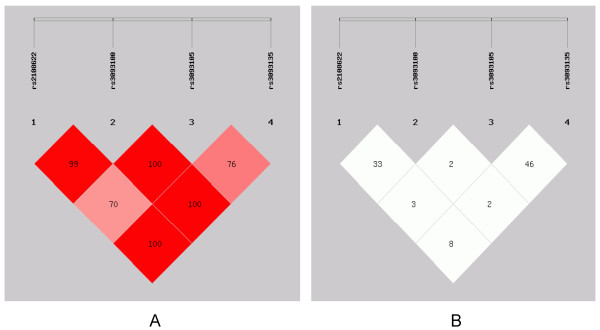
**Genetic variation at human CYP4F2 gene.** Using the SHEsis software, we calculated the Linkage disequilibrium (LD) value between each SNP. **A**: LD value shown: |D’| × 100; |D’| colour scheme: |D’| <0.5: white; 0.5 < r ^2^ < 1: shades of Pink; |D’| = 1: red; **B**: LD value shown: r ^2^ × 100; r ^2^ colour scheme: r ^2^ < 0.5: white; 0.5 < r ^2^ < 1: shades of grey; r ^2^ = 1: black.

According to the results of linkage disequilibrium analysis (As shown in Figure 1), we chose rs2108622-rs3093100-rs3093105-rs3093135 to construct haplotypes using SHEsis software. The results showed that, GGGT haplotype distribution frequency in the CHD group was significantly higher than that in the control group. Individuals carrying GGGT Haplotype had 4.367 times increased risk of CHD (OR = 4.367, 95% CI: 2.241 ~ 8.510; P < 0.001). However, the GGTA frequency was lower in CHD patients than that in the control subjects. Individuals carrying GGTA Haplotype had 0.450 times decreased risk of CHD (OR = 0.450, 95% CI: 1.111 ~ 7.777; P < 0.001), (Table 4).

**Table 4:** Haplotype distribution between case and control

**Haplotypes**	**CHD**	**Control**	**P**	**OR**	**95%CI**
A C T T	108.00(0.123)	121.00(0.137)	0.310	0.866	0.656 ~ 1.144
A G T T	128.00(0.145)	152.00(0.173)	0.095	0.804	0.622 ~ 1.039
G G G A	114.85(0.131)	86.11(0.098)	0.058	1.366	1.016 ~ 1.838
G G G T	46.14(0.052)	10.89(0.012)	<0.001	4.367	2.241 ~ 8.510
G G T A	21.4(0.020)	44.89(0.051)	<0.001	0.450	0.111 ~ 0.777

## Discussion

In this haplotype-based case–control study, we found GGGT haplotype carriers have higher risk but GGTA haplotype carriers have decreased risk for CHD in Han Chinese population. To the best of our knowledge, this is the first study to reveal the relation between CYP4F2 polymorphisms and CHD in Chinese Han population.

The genetic variation is the molecular basis of human genetic diversity. The T allele of CYP4F2 rs2108622 represents a missense mutation that results in the change of valine 433 to methionine (V433M). This change in the primary structure of CYP4F2 affects enzyme activity, leading to changes in drug metabolism, physiology, and pathophysiology. Cha et al. [22] through a genome-wide association study identified rs2108622 as a genetic determinant of warfarin responsiveness for Japanese. However, no association was found between this SNP and CHD by a genome-wide association study approach. CHD is caused by the interaction between environmental and genetic factors. In this study, an important human metabolic enzyme P450 gene family member- CYP4F2 gene was selected as the candidate gene to perform the case–control study, and we found that rs2108622 and rs3093105 in CYP4F2 gene was significantly associated with higher risk for CHD. We also found GGGT haplotype consisting of rs2108622-rs3093100-rs3093105-rs3093135 was the susceptibility haplotype of CHD. Our results were consistent with the results reported by Fu et al. [17], but inconsistent with results by Ward et al. [23] and Fava et al. [15]. Ward et al. found that the A allele was a risk factor for hypertension. The other study by Fava et al. [15] found that the A allele was a risk factor for IS. This difference may be due to different races, different methodologies and different patient selection criteria.

Singh et al. [24] found that androgen-induced CYP4A8 expression reduced CYP2C23 expression and caused increased production of 20-HETE, then the epoxyeicosatrienoic acid (EET) decreased to affect the contraction of blood vessels. However, the exact mechanism of the susceptibility for CHD remains unclear.

In addition, although an elevated level of LDL-C and a decreased level of HDL-C in serum are independent risk factors for CHD, we did not find the LDL-C level and HDL-C level to be different between the CHD patients and the control subjects. This phenomenon may be the result from the treatment of decreased-cholesterol drugs (e.g., simvastatin, lovastatin) in CHD patients.

Several limitations of the present study should be noted. Firstly, these findings should be interpreted with caution because the population was only from Chinese population, this fact may reduce the possibility of confounding from ethnicity; Secondly, the present study is a hospital based case–control study, the selection bias cannot be avoidable and the subjects may not be representative of the general population; Finally, in the present study, we did not detect the amount of 20-HETE associated with CYP4F2 polymorphisms.

## Conclusion

In conclusion, this study showed that both rs2108622 and rs3093105 in CYP4F2 gene were associated with CHD in Han Chinese population.

## Competing interests

The authors declared no competing interests exist.

## Authors’ contributions

CY and QYcarried out the molecular genetic studies and drafted the manuscript. CF and WS carried out the genotyping. HW, XW, and CZ participated in the design of the study and performed the statistical analysis. CY and CZ conceived of the study, and participated in its design and coordination and helped to draft the manuscript. All authors read and approved the final manuscript.
